# Determination of DNA methylation associated with *Acer rubrum* (red maple) adaptation to metals: analysis of global DNA modifications and methylation‐sensitive amplified polymorphism

**DOI:** 10.1002/ece3.2320

**Published:** 2016-07-22

**Authors:** Nam‐Soo Kim, Min‐Ji Im, Kabwe Nkongolo

**Affiliations:** ^1^ Department of Molecular BioscienceCollege of Biomedical ScienceKangwon National UniversityChuncheon200701Korea; ^2^ Institute of Bioscience and BiotechnologyCollege of Biomedical ScienceKangwon National UniversityChuncheon200701Korea; ^3^ Department of BiologyLaurentian UniversitySudburyONP3E‐2C6Canada

**Keywords:** *Acer rubrum*, DNA methylation, LC‐MS/MS, metal contamination, methylation‐sensitive amplified polymorphism, Northern Ontario

## Abstract

Red maple (*Acer rubum*), a common deciduous tree species in Northern Ontario, has shown resistance to soil metal contamination. Previous reports have indicated that this plant does not accumulate metals in its tissue. However, low level of nickel and copper corresponding to the bioavailable levels in contaminated soils in Northern Ontario causes severe physiological damages. No differentiation between metal‐contaminated and uncontaminated populations has been reported based on genetic analyses. The main objective of this study was to assess whether DNA methylation is involved in *A. rubrum* adaptation to soil metal contamination. Global cytosine and methylation‐sensitive amplified polymorphism (MSAP) analyses were carried out in *A. rubrum* populations from metal‐contaminated and uncontaminated sites. The global modified cytosine ratios in genomic DNA revealed a significant decrease in cytosine methylation in genotypes from a metal‐contaminated site compared to uncontaminated populations. Other genotypes from a different metal‐contaminated site within the same region appear to be recalcitrant to metal‐induced DNA alterations even ≥30 years of tree life exposure to nickel and copper**.** MSAP analysis showed a high level of polymorphisms in both uncontaminated (77%) and metal‐contaminated (72%) populations. Overall, 205 CCGG loci were identified in which 127 were methylated in either outer or inner cytosine. No differentiation among populations was established based on several genetic parameters tested. The variations for nonmethylated and methylated loci were compared by analysis of molecular variance (AMOVA). For methylated loci, molecular variance among and within populations was 1.5% and 13.2%, respectively. These values were low (0.6% for among populations and 5.8% for within populations) for unmethylated loci. Metal contamination is seen to affect methylation of cytosine residues in CCGG motifs in the *A. rubrum* populations that were analyzed.

## Introduction

Stable epigenetic variation might play an important role in plant adaptation and evolution. A number of publications have provided convincing evidence that abiotic stresses, such as DNA damage, drought, and high salinity, are involved in DNA methylation (Labra et al. 2002; Choi and Sano 2007; Peng and Zhang 2009; Chinnusamy and Zhu 2009; Verhoeven et al. 2010; Kimatu et al. 2011). These are in addition to biotic interactions and the mediators of biotic defense, interstrain or interspecies hybridization, as well as jumps in ploidy level (Mason et al. 2008; Li and Franke 2011; Richards 2011). Only the limited studies have been conducted under environmental conditions that plants may experience in real ecosystems outside the laboratory.

The Greater Sudbury Region (GSR) in Northern Ontario was one of the most ecologically disturbed regions in Canada, with numerous studies documenting the effects of SO_2_ and metals in soils in the region for >100 years (Cox and Hutchinson 1980; Amiro and Courtin 1981; Hutchinson and Symington 1997; Gratton et al. 2000; Nkongolo et al. 2008; Spiers et al. 2012). Elevated concentrations of metal accumulation have been reported in both soils and vegetation up to 100 km distant from the smelters compared to the reference sites and regional soil parent materials (Freedman and Hutchinson 1980; Gratton et al. 2000; Nkongolo et al. 2008; Dobrzeniecka et al. 2011; Vandeligt et al. 2011; Spiers et al. 2012). The presence of metal contaminants at elevated concentrations in the soil imposes a severe stress on plants, thus hindering the growth of vegetation (Wren 2012).

Red maple (*Acer rubum*) is a common deciduous tree species in Northern Ontario (Canada), including mining reclaimed areas in the GSR. It represents over 30% of all tree species in the region (Kalubi et al. 2015). This species has been reported to be fairly resistant to municipal landfill leachates (Gordon et al. 1989). In certain parts of Maryland, West Virginia, and Florida, seedlings have been observed to colonize strip mine spoils (Hardt and Forman 1989; Manci 1989).

**Figure 1:**
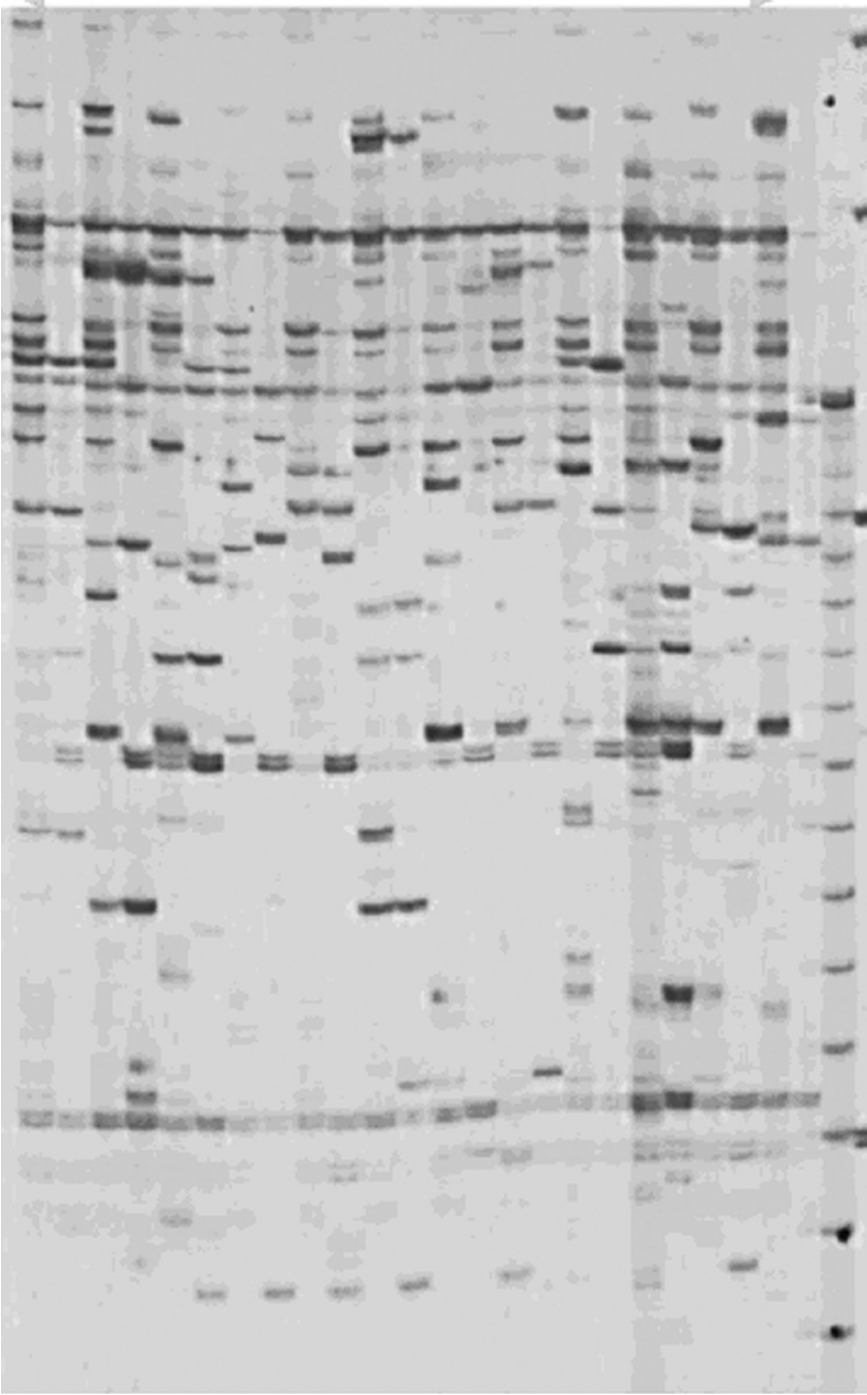
A methylation‐sensitive amplified polymorphism profile of an *Acer rubrum* population.

Genetic analysis of populations of *Acer rubrum* and other tree species growing in the GSR has been conducted and revealed no differences among metal‐contaminated and uncontaminated populations (Dobrzeniecka et al. 2011; Theriault et al. 2013, 2014; Tran et al. 2014; Kalubi et al. 2015). Unlike other hardwood species analyzed to date, *A. rubrum* does not accumulate metals in its leaves (Kalubi et al. 2016). This might be due to detoxification mechanisms used by plants to adapt to soil metal contamination. Analysis of DNA modifications in these populations is lacking. Epigenetic adaptability is an important and currently poorly understood factor in the survival and success of tree species in industrially contaminated areas. The main objective of this study was to assess whether DNA methylation is involved in *A. rubrum* adaptation to soil metal contamination in Northern Ontario (Fig. 1).

## Materials and Methods

### Sampling

**Figure 2:**
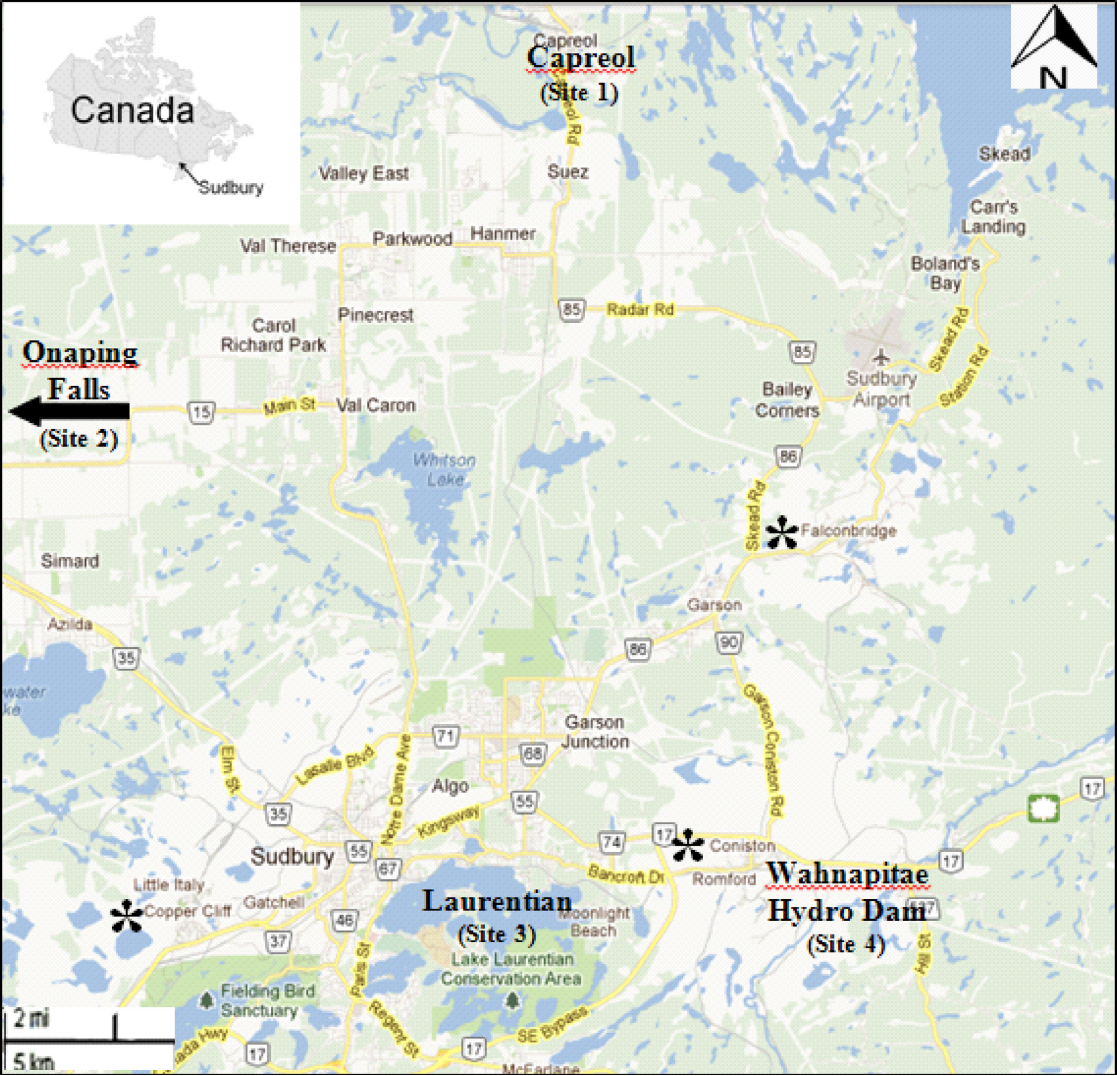
Location of sampling area from the Greater Sudbury Region. Site 1: Capreol (uncontaminated); Site 2: Onaping Falls Hydro Dam (uncontaminated); Site 3: Laurentian (metal‐contaminated); Site 4: Wahnapitae Hydro Dam (metal‐contaminated). 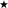 Represent smelter locations. *Sources: Edited from Google Map 2014*.

Four populations from the GSR in Northern Ontario were surveyed. Two were metal‐contaminated (Laurentian and Wahnapitae Hydro Dam), and two were reference sites‐uncontaminated (Capreol and Onaping Falls) (Fig. 2). For each site, 20 *A. rubrum* trees representing each targeted population were selected for the study. Leaves were collected from different branches for each individual tree. To avoid variability caused by biological samples, leaves were harvested from trees at the same developmental stages and between 25 and 30 years based on previous ecological studies (Kalubi et al. 2016). The samples were wrapped in aluminum foil, immersed in liquid nitrogen, and stored at −20°C until DNA extraction. Soil samples from the rhizosphere of each tree were also collected for metal analysis.

### Metal analysis

Metal analysis was performed only for soil samples because previous studies of the same sites revealed that *A. rubrum* does not accumulate metal in leaves (Kalubi et al. 2016). This analysis followed the method described by Nkongolo et al. (2013). For estimation of total metal concentration, 0.5 g of soil was treated with 10 mL of 10:1 ratio HF:HCl and heated to 110°C for 3.5 h in open 50‐mL Teflon^™^ tube in a programmable digestion block to dry down samples, followed by the addition of 7.5 mL of HCl and 7.5 mL of HNO_3_ and heating to 110°C for another 4 h to dry gently. The samples were then heated to 110°C for 1 h following the addition of 0.5 mL of HF, 2 mL of HCl, and 10 mL of HNO_3_ to reduce sample volume to 8–10 mL. Upon cooling, the samples were made to 50 mL with ultrapure water for subsequent analysis by plasma spectrometry. Bioavailable metals were estimated by extracting 5 g of soil with 20 mL of 0.01 mol/L LiNO_3_ in a 50‐mL centrifuge tubes in a shaker under ambient lighting conditions for 24 h at 20°C (Abedin et al. 2012). The pH (LiNO_3_) of the suspension was measured prior to centrifugation at 1000 g for 20 min, with filtration of the supernatant through a 0.45‐*μ*m filter into a 20‐mL polyethylene tube and made to volume with deionized water. The filtrate was preserved at approximately 3°C for analysis by ICP‐MS. The quality control program completed in an ISO 17025 accredited facility (Elliot Lake Research Field Station of Laurentian University) included analysis of duplicates, Certified Reference Materials (CRMs), Internal Reference Materials, procedural and calibration blanks, with continuous calibration verification, and use of internal standards (Sc, Y, Bi) to correct for any mass bias. All concentrations were calculated in mass/mass dry soil basis. The data obtained for all elements of interest in analyzed CRM soil samples were within ± 12% of the certified level.

### DNA extraction

Genomic DNA was extracted from frozen leaf material using the cetyl trimethylammonium bromide (CTAB) extraction protocol as described by Mehes et al. (2007). The protocol is a modification of the Doyle and Doyle (1987) procedure. The modifications included the addition of 1% polyvinyl pyrrolidone and 0.2% beta mercaptanol to the CTAB buffer solution, two additional chloroform spins prior to the isopropanol spin, and no addition of RNAse. After extraction, DNA was stored in a freezer at −20°C.

### Global DNA methylation

The general protocol is described in Tsuji et al. (2014). Nucleoside quantification was determined using tandem mass spectrometry (MS/MS) coupled with LC (LC‐MS/MS). Genomic DNA was digested with DNA Degradase Plus (ZYMO RESEARCH, Irvine, CA) per the manufacturer protocol. LC separation was performed on a dC18 2.1 × 100 mm column at a flow rate of 0.2 mL/min. The mobile phase was 15% CH_3_OH, 85% H_2_O with 1% formic acid, and 10 mmol/L ammonium formate. The injection volume was 15 μL. A Waters/Micromass Quattro Micro mass spectrometer was used for the detection of nucleosides. Electrospray ionization in positive ion mode was used to generate ions. Methylation levels are reported as [5 mdC]/[dG], ratio.

Data from 20 trees for each population were analyzed using SPSS 20 for Windows, with all values being log_10_ transformed to achieve a normal distribution. ANOVA, followed by Tukey's HSD multiple comparison analysis, was performed to determine the significant differences in metal content and global cytosine methylation (*P* ≤ 0.05) among populations.

### Methylation‐sensitive amplified polymorphism analysis

The protocol of MSAP was adapted from those of Reina‐López et al. (1997) and Xiong et al. (1999). Two isoschizomers, *Hpa*II and *Msp*I, were employed for cutting methylation‐sensitive site CCGG. The genomic DNA was restriction‐digested with either *EcoR*I/*Hpa*II or *Eco*I/*Msp*I. The digested DNA was ligated with linkers at both ends and then PCR‐amplified with primers having one selective nucleotide at 3′‐end. Then, selective amplification was carried out with eight primer pairs (EA‐ATG/HM‐GCA, EA‐ATG/HM‐TCT, EA‐ATG/HM‐CAT, EA‐CGT/HM‐GCA, EA‐CGT/HM‐TCT, EA‐CGT/HM‐CAT, EA‐CGC/HM‐GCA, and EA‐CGC/HM‐CAT). The final products were separated in 6% denaturing PAGE at 1800 volts at 50 watts for 150 min. The separated DNAs were visualized by silver staining (Promega, Madison, WI). Nucleotide sequences of the adaptors and primers are enlisted in the Table 1.

**Table 1:** Primers used for methylation‐sensitive amplified polymorphism analysis and their sequences

Primers	Sequence (5′→3′)
E‐0	GACTGCGTACCAATTC
EA‐1	AATTGGTACGCAGTCTAC
EA‐2	CTCGTAGACTGCGTACC
EA‐ATG	GACTGCGTACCAATTATG
EA‐CGT	GACTGCGTACCAATTCGT
EA‐CGC	GACTGCGTACCAATTCGC
HM‐0	CATGAGTCCTGCTCGG
HM‐1	GATCATGAGTCCTGCT
HM‐2	CGAGCAGGACTCATGA
HM‐GCA	CATGAGTCCTGCTCGGGCA
HM‐TCT	CATGAGTCCTGCTCGGTCT
HM‐CAT	CATGAGTCCTGCTCGGCAT

Scoring of MSAP was also done either 1 or 0 by the presence or absence of amplicons in both *Hpa*II and *Msp*I. However, interpretation of polymorphism was modified because sensitivity of *Hpa*II and *Msp*I was different depending on the cytosine methylation status at CCGG. Bands of *Hpa*II and *Msp*I were scored in each samples as 1/1 (bands present in both enzyme digestions), 1/0 (band present in *HpaII*, but absent in *MspI*), 0/1 (band absent in *HpaII*, but present in *MspI*), or 0/0 (band absent for both enzyme digestions). For verification of homologous bands in each lane, the bands at the corresponding positions were isolated for sequence verifications.

Genetic variations were analyzed using POPGENE version 1.32 (https://www.ualberta.ca/~fyeh/popgene.html). Polymorphic information content (PIC) and genetic distances among populations were analyzed using POWERMARKER version 3.25 (http://statgen.ncsu.edu/powermarker/), and genetic distances were used for UPGMA genetic dendrogram construction using MEGA5 (Tamura et al. 2011). MSAP‐R computer software (http://cran.r-project.org) was used for the calculation of genetic (no‐methylated loci; NML) and epigenetic (methylation‐susceptible loci; MSL) variations from principal coordinate analysis (PCA) data, which were then used for neighbor‐joining tree construction for population differentiation by genetic versus epigenetic variations (Pérez‐Figueroa 2013).

## Results

### Metal analysis

There were significantly higher levels of total copper (Cu) and nickel (Ni) in samples from sites close to smelters compared to reference (uncontaminated) sites (Table 2). No statistical differences for metal contamination between the two metal‐contaminated sites and between the two reference sites for Ni and Cu were observed. The proportion of total metal and/or nutrient that was phytoavailable was determined with the analytical results revealing that the bulk of the total metal and/or nutrient in the soil matrix is not in forms either on the exchange surface of mineral or organic colloids or in the soil solution available for plant uptake (Table 2).

**Table 2:** Total and bioavailable metal concentrations (in parentheses) from four sites in the Greater Sudbury Region (Concentration in mg/kg)

Sites	Copper (Cu)	Nickel (Ni)	Zinc (Zn)
Metal‐contaminated			
Wahnapitae Dam	890.00a (19.60)	2030.00b (12.90)	109.00a (2.19)
Laurentian	2020.00a (14.20)	3010.00a (10.40)	147.00a (1.46)
Reference			
Onaping Falls (Control)	110.00b (0.00)	165.00c (0.00)	85.80a (0.19)
Capreol (Control)	188.00b (2.09)	259.00c (2.47)	96.80a (3.37)

Means values with the same letter within each dataset (Cu, Ni, and Zn) are not significantly different based on Tukey's HSD multiple comparison test (*P* ≥ 0.05; *n* = 20).

### Global cytosine methylation

**Figure 3:**
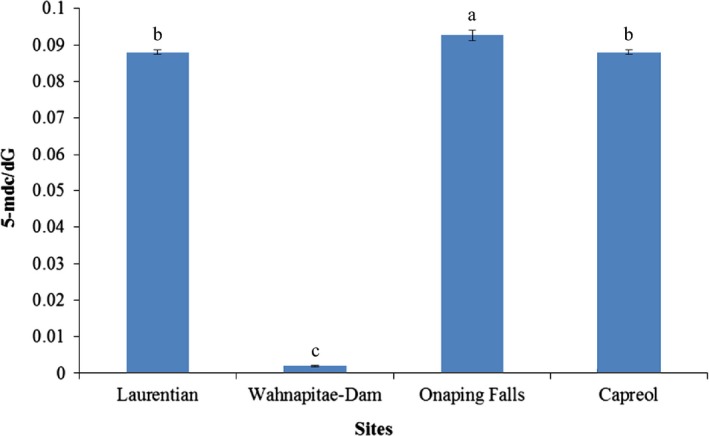
Global DNA methylation levels [(5‐mdC)/dG] in DNA from *Acer rubrum* trees from Laurentian (metal‐contaminated), Wahnapitae Dam (metal‐contaminated), Onaping Falls (reference), and Capreol (reference). Means values with the same letter within each dataset (Cu, Ni, and Zn) are not significantly different based on Tukey's HSD multiple comparison test (*P* ≥ 0.05; *n* = 20). Bars represent standard errors.

The extent of cytosine methylation in DNA was assessed by the ratio of 5‐methyldeoxycytidine (5 mdC) to deoxyguanosine (dG). There was a significant difference in the level of cytosine methylation between the two metal‐contaminated sites (Fig. 3). A lower level of cytosine methylation was observed in samples from Wahnapitae Hydro Dam compared to the other three populations that exhibited similar levels of methylation: The mean levels of [(5‐mdC)/dG] were 0.002 (0.02%) for Wahnapitae Hydro Dam (contaminated), 0.088 (8.8%) for Laurentian (contaminated), 0.088 (8.8%) for Capreol (uncontaminated), and 0.091 (9.1%) for Onaping (uncontaminated). Overall, the average levels of cytosine methylation were 0.045 (4.5%) and 0.09 (9.0%) for metal‐contaminated sites and uncontaminated sites, respectively. Genotypes from the Laurentian site could be recalcitrant to cytosine methylation as the levels of [(5‐mdC)/dG] were similar to those in uncontaminated sites.

### Methylation‐sensitive amplified polymorphism analysis

The number of MSAP loci for each primer combination ranged from 20 to 30 between 150 and 600 bp. Overall, a total of 205 loci were detected of which 127 were methylation‐susceptible (MSL) and 78 nonmethylated (NML). The MSL were 1/0, 0/1, and 0/0 in band reading for *Hpa*II and *Msp*I digestions. The NML were 1/1 in all plants. The nucleotide sequences of the corresponding position in the gel revealed that co‐migrating bands at the same positions were homologous sequence by identical sequences (Fig. S1). Detailed analysis shows that 6.8% of loci were monomorphic nonmethylated, 5.9% monomorphic CpG methylated, 4.4% polymorphic nonmethylated, 7.3% polymorphic CpG methylated, 5.4% polymorphic CpCpG methylated, and 70.2% polymorphic hypermethylated. The levels of polymorphism were 68% and 72% for MSL and NML, respectively. MSAP profile is shown in the Figure S2.

**Figure 4:**
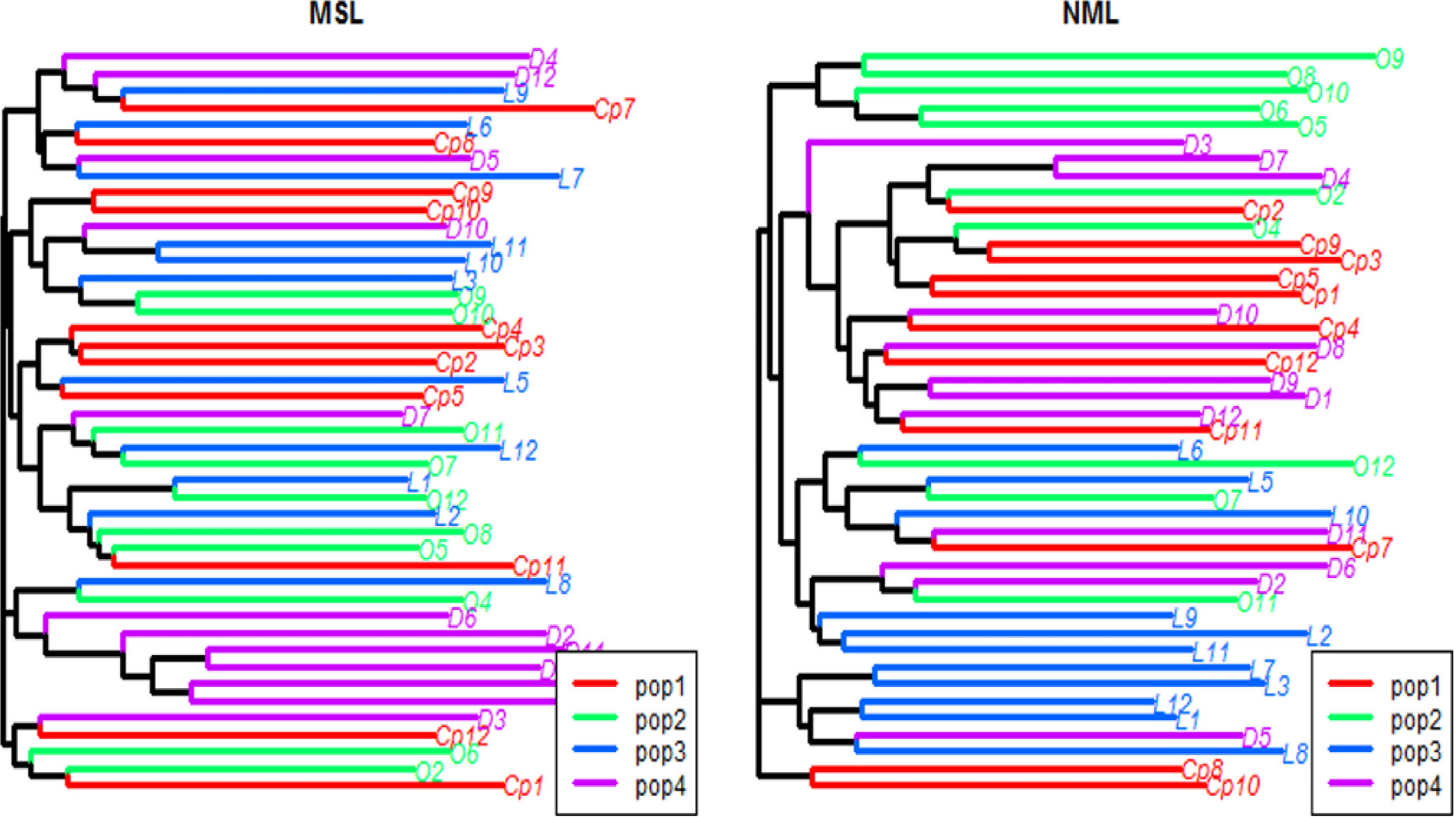
Clustering of *Acer rubrum* genotypes based on methylation‐sensitive amplified polymorphism data using MEGA 5 program. Cp represents genotypes from Capreol, O from Onapings Fall, L from Laurentian, and D from Wahnapitae Hydro Dam. General genotype grouping consistent with the site location. P1, P2, P3, and P4 represent Capreol, Onaping, Laurentian, and Dam sites, respectively.

**Figure 5:**
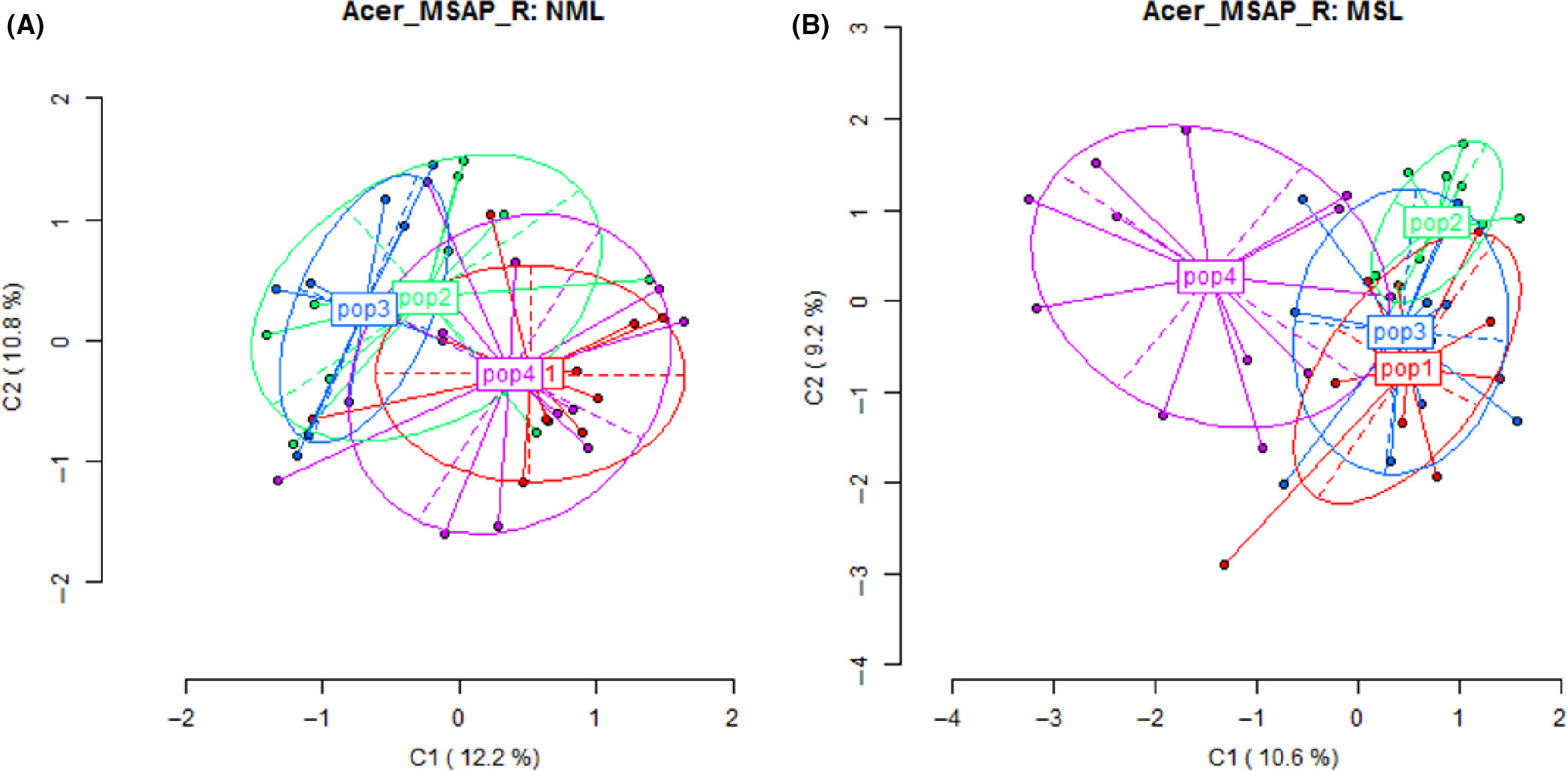
Principal component analysis (PCA) of *Acer rubrum* populations (Pop 1 and Pop 2 are uncontaminated, and Pop 3 and Po4 are metal‐contaminated). (A) Nonmethylated loci and (B) methylated loci. Percentages indicated the proportion of variation explained by each ordination. P1, P2, P3, and P4 represent Capreol, Onaping, Laurentian, and Dam sites, respectively.

No specific methylated or demethylated locus was observed in the metal‐contaminated or uncontaminated (reference) populations. Thus, very little population differentiation between samples from uncontaminated and metal‐contaminated groups was observed based on the level of polymorphism, observed and effective number of alleles, Nei's gene diversity (*I *=* *1 − $\sum_{i=1}^{m}x_{i}^{2}$ in which *x*_*i*_ is the frequency of allele *i*, and *m* is the number of alleles that have found at the locus), and Shannon's information index ($H^{\prime }=-\sum_{i=1}^{R}p_{i}\text{ln}p_{i}$, in which *p*_*i*_ is the proportion of alleles belonging to the *i*th type in the dataset) (Nei 1973; Begon et al. 1996). The percentage of polymorphic loci was 77% with the PIC of 0.1745 for uncontaminated populations. These values were 72% for polymorphism and 0.1728 for PIC for metal‐contaminated populations (Table 3). The NJ dendrograms based on the genetic variations of MSL and NML revealed that the populations were admixed in both dendrograms. But individuals within the same population tend to cluster together in dendrograms (Fig. 4). The principal component analyses (PCA) of the Methylation sensitive amplified Polymorphism (MSAP) profile did not separate the groups by the levels of metal contamination because there were significant overlaps among populations (Fig. 5). But, the PCA scores for methylated loci showed that Wahnapitae Hydro Dam population was partially separated from the other three populations (Laurentian, Capreol, and Onaping) (Fig. 5).

**Table 3:** Genetic parameters from the amplification of DNA from metal‐contaminated and uncontaminated sites using methylation‐sensitive primers

Genetic parameters	Uncontaminated	Metal‐contaminated
Number of primer sets	8	8
Number of loci per primer set	46	46
Number of polymorphic loci	284	266
Percentage of polymorphic loci	76.55	71.70
Observed number of alleles/epialleles per locus (na)	1.7655 ± 0.42	1.717 ± 0.45
Effective number of alleles/epialleles per locus (ne)	1.3264 ± 0.33	1.3254 ± 0.34
na‐ne	0.4391	0.3916
Nei's (1973) gene diversity (He)	0.2026 ± 0.18	0.2 ± 0.18
Shannon's information index (I)	0.3183 ± 0.25	0.3112 ± 0.26
Mean genetic/epigenetic distance	0.2405	0.2395
Minimum genetic/epigenetic distance	0.0000	0.0000
Maximum genetic/epigenetic distance	0.3868	0.3632
Relative degree of genetic/epigenetic diversity (Gst)	0.0865	0.0962
Gene flow (Nm)	5.2807	4.698
Polymorphic information content (PIC)	0.1745	0.1728
Neutrality	0.734762	0.740847
Number (%) of non‐neutral sites	0.265238	0.259153

The variations for nonmethylated and methylated loci were compared by AMOVA. Overall, for methylated loci, the molecular variance among and within populations was 1.5% and 13.2%, respectively. These variance values were 0.6% (among populations) and 5.8% (within populations) for NML (Table 4). Thus, metal contamination affects methylation of cytosine residues in CCGG motifs in *A. rubrum* populations analyzed. However, no population‐specific (methylated or no‐methylated) locus was identified in the sample set.

**Table 4:** Analysis of molecular variance for the (a) methylated and (b) nonmethylated loci

Parameters	df	SSD	MSD	Variance
(a)				
Among groups	3	91.47	30.49	1.577
Within groups	40	526.6	13.17	13.17
Total	43	618.1	14.37	
(b)				
Among groups	3	36.31	12.1	0.568
Within groups	40	234.6	5.864	5.864
Total	43	270.9	6.3	

(a): Phi‐ST = 0.107 (*P* < 0.0001).

(b): Phi‐ST = 0.0883 (*P* < 0.0001).

## Discussion

### Analysis of cytosine methylation

Previous and present soil chemistry analyses revealed that the uncontaminated and metal‐contaminated sites targeted in the present study have similar levels of soil acidity, cation exchanges, and soil nutrients, but showed significant difference only for Ni and Cu content (Nkongolo et al. 2013; Kalubi et al. 2015, 2016). Although the levels of total metals (Ni, Cu, and Zn) were high in contaminated sites, the bioavailable portion was small as reported in the previous studies (Nkongolo et al. 2013). It should be noted that the levels of bioavailable elements detected can vary based on the extraction methods. In fact, the levels of bioavailable Ni, Cu, and Zn found in the present study are lower compared to data described in Mehes‐Smith and Nkongolo (2015) using a different metal extraction procedure. Recent studies have demonstrated that the levels of bioavailable metals in the GSR soils cause overtime significant physiological damages to plants (Tanentzap and Ryser 2015). These authors showed that *A. rubrum* saplings from within the metal‐contaminated sites in the GSR had twice the percent loss of conductivity, caused by embolisms, as compared to saplings from the uncontaminated areas. Moreover, the bioavailable levels of metals in the GSR caused a decrease in stomatal density and chlorophyll content in *A. rubrum* (de Silva et al. 2012). These effects are amplified when metal contaminations are combined with drought leading to reductions in hydraulic conductance, xylem‐specific conductivity, and leaf‐specific conductivity (de Silva et al. 2012).

It is also established that *A. rubrum* populations colonizing the region are from the same surrounding areas. Considering that the targeted trees were between 30 and 35 years old, the effects of long‐term exposure to metals such as hypomethylation or hypermethylation of DNA were expected based on the previous studies on stress‐induced methylation (Choi and Sano 2007; Wang et al. 2011; Shan et al. 2013). In fact, in rice (*Oryza sativa*) it has been demonstrated that drought stress causes changes in genomic methylation levels (Wang et al. 2011). Likewise, DNA methylation increases under salt stress in tobacco, and it decreases under cold stress in maize (*Zea mays*) (Choi and Sano 2007; Shan et al. 2013). High levels of aluminum in soil and low pH have been linked to DNA modifications caused by methylation in *Sorghum bicolor* (Kimatu et al. 2011).

Two methods were used to measure the levels of cytosine methylation in the present study. Tandem mass spectrometry (MS/MS) coupled with LC (LC‐MS/MS) is an established approach to nucleoside quantification specifically to measure global cytosine methylation (Hu et al. 2013; Tsuji et al. 2014). In particular, it is a fast, sensitive, accurate, and specific avenue for modified nucleoside quantification at trace (fmol) levels (Alonso et al. 2016). Quantitative data revealed significant decrease in cytosine methylation in metal‐contaminated sites especially at the Wahnapitae Hydro Dam site. However, genotypes growing on Laurentian site appear to be recalcitrant to cytosine modification.

For the quantification of genome‐wide cytosine methylation, the chemical method (LC‐MS/MS) used in this study is the most recommended because of its global assessment, accuracy, and reproducibility (Fraga and Esteller 2002; Lisanti et al. 2013; Alonso et al. 2016). MSAP was also performed to determine the differentiation in cytosine methylation in targeted populations. Although the scoring of MSAP markers is not straightforward, the technique does not require a sequenced reference genome and provides many anonymous loci randomly distributed over the genome. Moreover, methylation detected by MSAP is greater than genome‐wide estimates obtained by HPLC (Alonso et al. 2016). The combination of both techniques (MSAP and HPLC) provides complementary information that improves our understanding of DNA methylation. The MSAP analysis revealed no significant differences between uncontaminated and metal‐contaminated populations for all the genetic parameters tested in the present study. But cytosine methylation was shown to be partitioned, with different patterns between populations versus within populations of *Acer rubrum*. The results showed that methylated loci are more variable than nonmethylated loci in both metal‐contaminated and uncontaminated populations. PCA revealed that *A. rubrum* population from Wahnapitae Hydro Dam was partially separated from other populations based on the methylated loci. This is consistent with global DNA methylation variation observed among the four populations.

It should be pointed out that there is a wide range of techniques in addition to the two methods used in this study that are available to characterize methylcytosine in a genome (Saluz and Jost 1993; Grigg and Clark 1994; Rein et al. 1998; Fraga and Esteller 2002; Laird 2010; Alonso et al. 2016; Herrera et al. 2016; Schrey et al. 2013). Each technique has its own peculiarities. In general, the earliest approaches studied the overall levels of methylcytosine. But more recent efforts have concentrated on the analysis of the methylation status of specific DNA sequences (Fraga and Esteller 2002). Optimization of the methods based on bisulfite modification of DNA facilitates the analysis of limited CpGs in restriction enzyme sites and the overall characterization based on differential methylation and allows very specific patterns of methylation to be revealed (bisulfite DNA sequencing) (Shen and Waterland 2007). Moreover, new techniques designed to search for new methylcytosine hot spots have significantly contributed to our understanding of DNA alterations without requiring prior knowledge of the genome sequence (Fraga and Esteller 2002; Shen and Waterland 2007).

### Effects of metals on DNA methylation

Studies in herbaceous plant species showed that heavy metals (nickel, cadmium, and chromium) induced a global dose‐dependent decrease of 5 mC content ranging from 20% to 40% in *Trifolium repens* L. and *Cannabis sativa* L (Aina et al. 2004). These effects might be underestimated since the authors used slot blot hybridization: a less sensitive method compared to LC (LC‐MS/MS) to measure cytosine methylation. Kovalchuk et al. (2003) reported an increase in global DNA methylation caused by radioactive contamination. Plant methylation in responses to stress was variable, and it depended on the type and level of stress, plant tissues, age, and species as documented by a number of reports (Labra et al. 2002; Aina et al. 2004; Peng and Zhang 2009; Rico et al. 2013).

Because of high cost associated with bisulfite sequencing, MASP has been often used to assess methylcytosine changes in ecological studies. It provides many anonymous loci randomly distributed over the genome for which the methylation status can be ascertained (Alonso et al. 2016). MASP is a robust, highly reproducible, and chip‐based molecular tool useful to detect variations in the DNA methylation status. Aina et al. (2004) reported hypomethylation of DNA induced by heavy metals, while Greco et al. (2012) observed a hypermethylation induced by cadmium in *Posidonia oceanica* based on MASP. Cicatelli et al. (2014) using the same technique showed an extensive DNA hypermethylation in leaves when mycorrhizal poplar (*Populus alba*) plants were grown in the presence of 950 mg/kg of total Zn and 1300 mg/kg of total Cu compared to control.

One of the shortcomings of this method that is a concern for the present study and others using MASP method is that some methylated states can be missed because the banding pattern observed when MsPI and HpaII fail to cut can be caused by genetic or epigenetic process. Also, differences between MspI and HpaII reactions could have been the result of inconsistent restriction digests or variation in PCR rather than by differential methylation. Moreover, there is no consensus on the interpretation and scoring of the four possible outcomes that can be obtained from the combined MSAP banding patterns (Fulnecek and Kovarik 2014). In the present study, the combination of global methylation and MASP analysis indicated that in addition to metals, site specificity is an important factor in plant epigenetic response to stress. This means that DNA modification results from factors other than soil chemistry and plant development. More investigations are needed to better understand the mechanisms involved in plant DNA modifications.

In animal models, hypermethylation caused by exposure to nickel compounds has been observed in hamster G12 cell line and in nickel‐induced tumors in wild‐type C57BL/6 mice (Lee et al. 1995; Arita and Costa 2009). It has been suggested that the ability of Ni to substitute for magnesium in the phosphate backbone of DNA might be the main mechanism leading to hypermethylation. Ni might be better at increasing chromatin condensation and triggering *de novo* DNA methylation of critical genes that can be incorporated into heterochromatin in animal models (Arita and Costa 2009). The fact that heterochromatin forms the inside lining of the interface nucleus and encounters toxins entering the nucleus before they reach euchromatin makes them the primary target of nickel ions (Conway et al. 1987; Costa et al. 1991; Arita and Costa 2009). Reports on cytosine methylation and metal‐induced stress in woody trees are limited. The mechanism of DNA alterations caused by metals in plants is unknown, but the lack of significant changes in DNA methylation in *Acer rubrum* plants exposed to nickel and copper contamination for over 30 years could be the result of a possible internal mechanism involved in metal ions processing in cells. In fact, plants can cope with metal through the sophisticated homeostatic cellular mechanisms that self‐regulate the content of metal ions in the cells to limit the negative impacts that result from exposure to nonessential metal ions (Hossain and Komatsu 2013). These mechanisms are used efficiently by some plant species to control metal uptake, mobilization, translocation, and detoxification. The plasma membrane exclusion system and the synthesis of membrane transporters and thiol‐containing chelating compounds for vacuolar sequestration are the main methods used by plants to protect the cell from the adverse effects of metals. In addition, defense protein and molecular chaperones help plants growing in metal‐contaminated soil to maintain redox homeostasis (Hossain and Komatsu 2013).

Nickel, copper, and zinc bioaccumulation and translocation processes in *A. rubrum* likely play a role for this limited effect of metals on DNA methylation in leaves in some genotypes. Previous reports revealed that the translocation factors of these elements are very small in *A. rubrum* and high in other hardwood trees such as *Populus tremuloides* and *Quercus rubra* (Tran et al. 2014; Kalubi et al. 2015, 2016; Mehes‐Smith and Nkongolo 2015). It is expected that the expression of genes involved in metal avoidance and exclusion is variable within segregating and heterozygous *A. rubrum* populations targeted in the present study.

## Conclusion

The levels of DNA methylation were analyzed in 25‐ to 30‐year‐old *A. rubrum* trees from metal‐contaminated and uncontaminated sites. A significant decrease in cytosine methylation was observed in a metal‐contaminated population, but genotypes recalcitrant to DNA modifications were prevalent in one of the metal‐contaminated populations even after a long‐term exposure to Ni and Cu based on LC‐MS/MS analysis. MASP analysis revealed a higher molecular variance for methylated loci compared to nonmethylated loci. Overall, Ni and Cu contamination affects methylation of cytosine residues in CCGG motifs in *A. rubrum* populations analyzed. Efficient cellular mechanisms might be involved in processing metal in *A. rubrum* cells that limit the impacts of metal ions on cytosine methylation in some genotypes.

## Conflict of Interest

None declared.

## Supporting information


**Figure S1**. Gel images of the homologous bands isolated (arrowhead pointed) in A and nucleotide sequence comparisons of the homologous bands in B.

**Figure S2**. MSAP profiles among the *A. rubrum* populations using different primer combinations.
