# Supplementary material for: Determination of DNA methylation associated with Acer rubrum (red maple) adaptation to metals: analysis of global DNA modifications and methylation‐sensitive amplified polymorphism
Source: Ecol Evol. 2016 Jul 22;6(16):5749–60. doi: 10.1002/ece3.2320 (PMC4983588; doi:10.1002/ece3.2320)
Supplement: Supplementary file 1 — Figure S1. Gel images of the homologous bands isolated (arrowhead pointed) in A and nucleotide sequence comparisons of the homologous bands in B. [file ECE3-6-5749-s001.pdf]

A

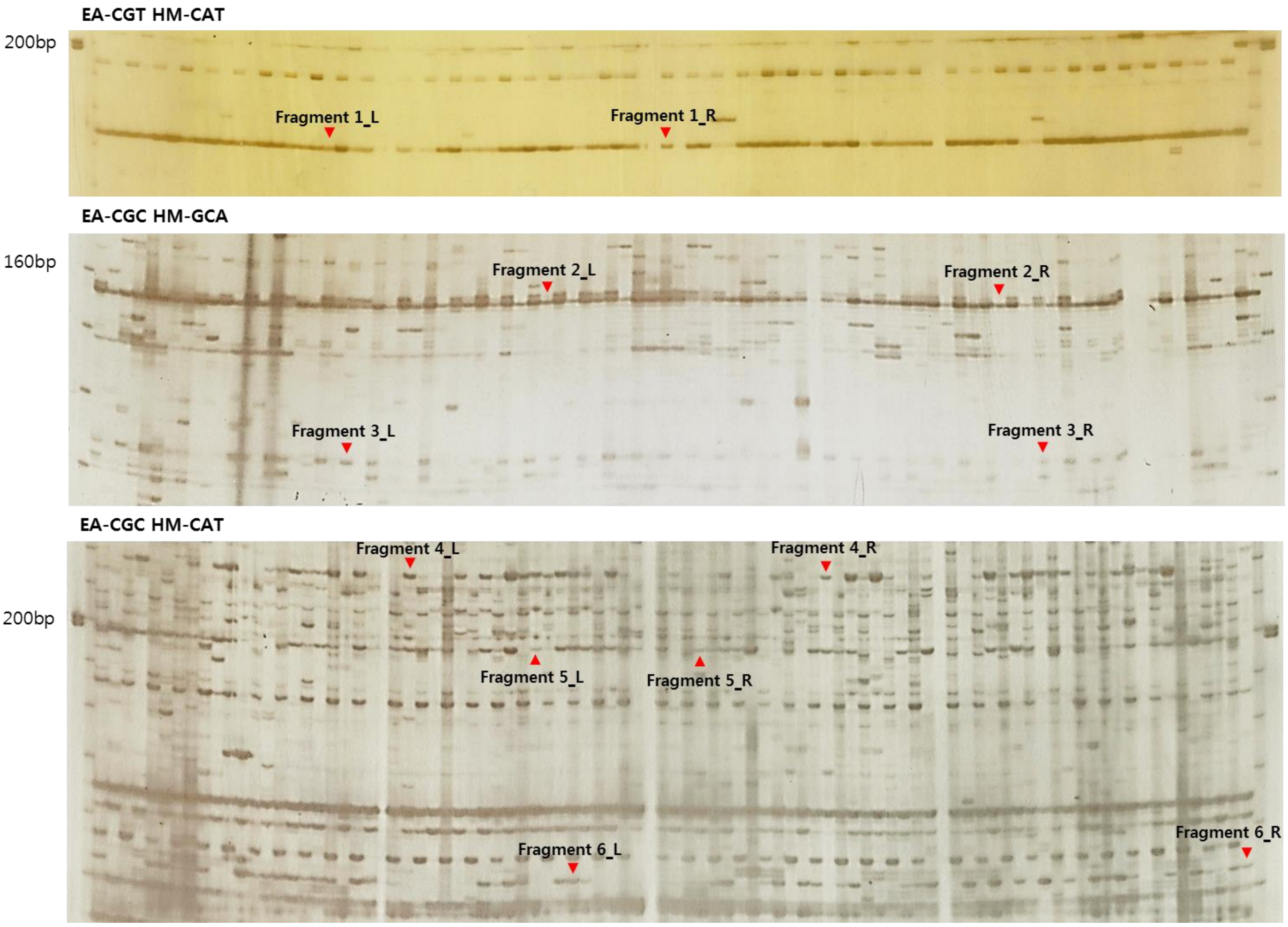

B

EA-CGT HM-CAT

> Fragment 1

```
L GACTGCGTACCAATTCCGTTTGGTGATGATCACAAGGGTTGCACAATACTTCTTACTACTCGTCAACAAGAAGTATGCAAAA
R GACTGCGTACCAATTCCGTTTGGTGATGATCACAAGGGTTGCACAATGCTTCTTACTACTCGTCAACAAGAAGTATGCAAAA
  ***********************************************.**********************************

L GTATGAGGTGTCAGCTGCAGATTCAATTAGATATTCTAAA
R GTATGAGGTGTCAGCCGCAGATTCAATTAGATATTCTAAA
  ***************.************************
```

EA-CGC HM-GCA

> Fragment 2

```
L CATGAGTCCTGCTCGGGCACCCACTACCAATCCGAAGGCGACGTGTGTTGTATTTTTGGTGGGAGATTCCCTTCTACTAC
R CATGAGTCCTGCTCGGGCACCCACTACCAATCCGAAGGCGACGTGTGTTGTATTTTTGGTGGGAGATTCCCTTCTACTAC
  ********************************************************************************

L ATTTCTATATTTAGACCTCTCGTATAACGGTATAAGCATTGTTTGGGGAAAAGCCACGCGGAATTGGTACGCAGTC
R ATTTCTATATTTAGACCTCTCGTATAACGGTATAAGCATTGTTTGGGGAAAAGCCACGCGGAATTGGTACGCAGTC
  ****************************************************************************
```

> Fragment 3

```
L CATGAGTCCTGCTCGGGCAATGGAGTCGGAATGATGTAAAACTTTCACAGTTGATGCTTTTCGGCGAGATCTTCAAAACT
R CATGAGTCCTGCTCGGGCAATGGAGTCGGAATGATGTAAAACTTTCACAGTTGATGCTTTTCGGTGAGATCTTCAAAACT
  ****************************************************************.***************

L CACAACCTTTGGTTGTGTGGCATGCCCTTTTTTAGCGGAATTGGTACGCAGTC
R CACAACCTTTGGTTGTGTGGCATGCCCTTTTTTAGCGGAATTGGTACGCAGTC
  *****************************************************
```

EA-CGC HM-CAT

> Fragment 4

```
L CATGAGTCCTGCTCGGCATGGGCATCTGCCGTGTCGCGTCCCAAAACGACTCTAAAATATATCCCTTAAATGATGCCTAGAAT
R CATGAGTCCTGCTCGGCATGGGCATCTGCCGTGTCGCGTCCCAAAACGACTCTAAAATATATCCCTTAAATGATGCCTAGAAT
  ***********************************************************************************

L AATTAAAATCTGTGTGAAAGCCTCCAACAATTAAAATGTGCAAAGTTAAAAGCCAAATAAGGCTTAGAGGATTTTTGTAAA
R AATTAAAATCTGTGTGAAAGCCTCCAACAATTAAAATGTGCAAAGTTAAAAGCCAAATAAGGCTTATAGGATTTTTGTAAA
  ******************************************************************.**************

L TTTCCTAAAAGCCTAAAATGCGTAAATAAATTTTAGCGGAATTGGTACGCAGTC
R TTTCCTAAAAGCCTAAAATGCGTAAATAAATTTTAGCGGAATTGGTACGCAGTC
  ******************************************************
```

> Fragment 5

```
L CATGAGTCCTGCTCGGCATAGATCTCGGGCGGGGATGGGATGGCAGAGGCGAGATGGATCGCCGACGATTTCCTCTTCTTTTT
R CATGAGTCCTGCTCGGCATAGATCTCGGGCGGGGATGGGATGGCAGAAGCGAGATGGATCGCCGACGATTTCCTCTTCTTTTT
  ***********************************************.***********************************

L CTCTCTGTTTTTCTTTTTCTTTAATTAAATCGGAAATCCAAAAACCCCTCCACACAGGGTTTACAATTATGACATGCCGATCCGTTT
R CTCTCTGTTTTTCTTTTTCTTTAATTAAATCGGAAATCCAAAAACCCCTCCACACAGGGTTTACAATTATGACATGCCGATCCGTTT
  ***************************************************************************************

L CAGACACTTGCGGAATTGGTACGCAGTC
R CAGACACTTGCGGAATTGGTACGCAGTC
  ****************************
```

> Fragment 6

```
L CATGAGTCCTGCTCGGCATGGACGAGGCCCAATATGGTAGCGATCCATCTCAGTGTGGGCCTCTAGTTAAATCAATGGGCCGTCAAGATTGGA
R CATGAGTCCTGCTCGGCATGGACGAGGCCCAATATGGTAGCGATCCATCTCAGTGTGGGCCTCTAGTTAAATCAATGGGCCGTCAAGATTGGA
  *********************************************************************************************

L CCCACGTTTCGGCCGTCAATTTCTTTTGCGGGCTGAATGGGCGGAATTGGTACGCAGTC
R CCCACGTTTCGGCCGTCAATTTCTTTTGCGGGCTGAATGGGCGGAATTGGTACGCAGTC
  ***********************************************************
```
