# Supplementary material for: Determination of DNA methylation associated with Acer rubrum (red maple) adaptation to metals: analysis of global DNA modifications and methylation‐sensitive amplified polymorphism
Source: Ecol Evol. 2016 Jul 22;6(16):5749–60. doi: 10.1002/ece3.2320 (PMC4983588; doi:10.1002/ece3.2320)
Supplement: Supplementary file 2 — Figure S2. MSAP profiles among the A. rubrum populations using different primer combinations. [file ECE3-6-5749-s002.pdf]

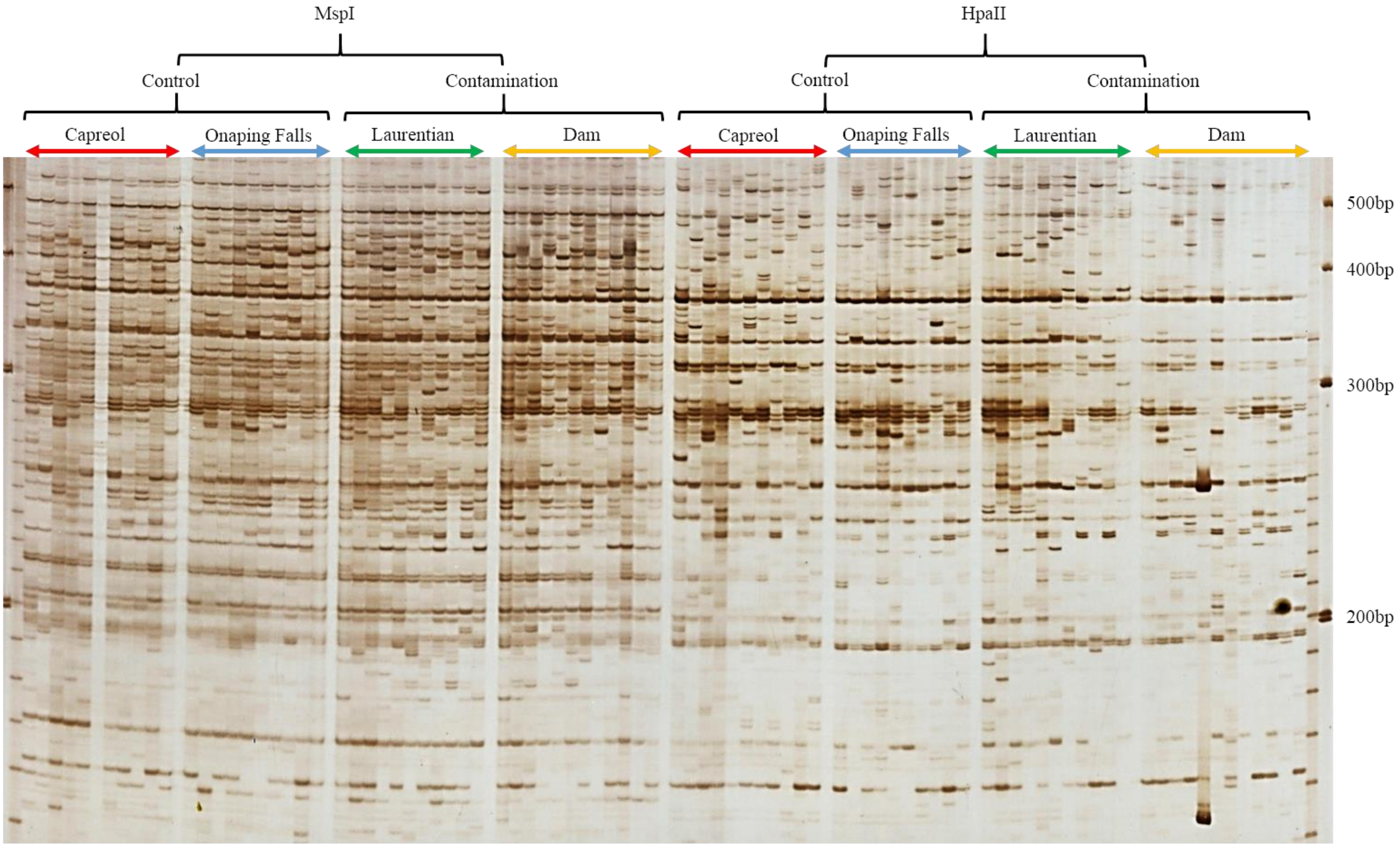

Supplementary Figure 2a

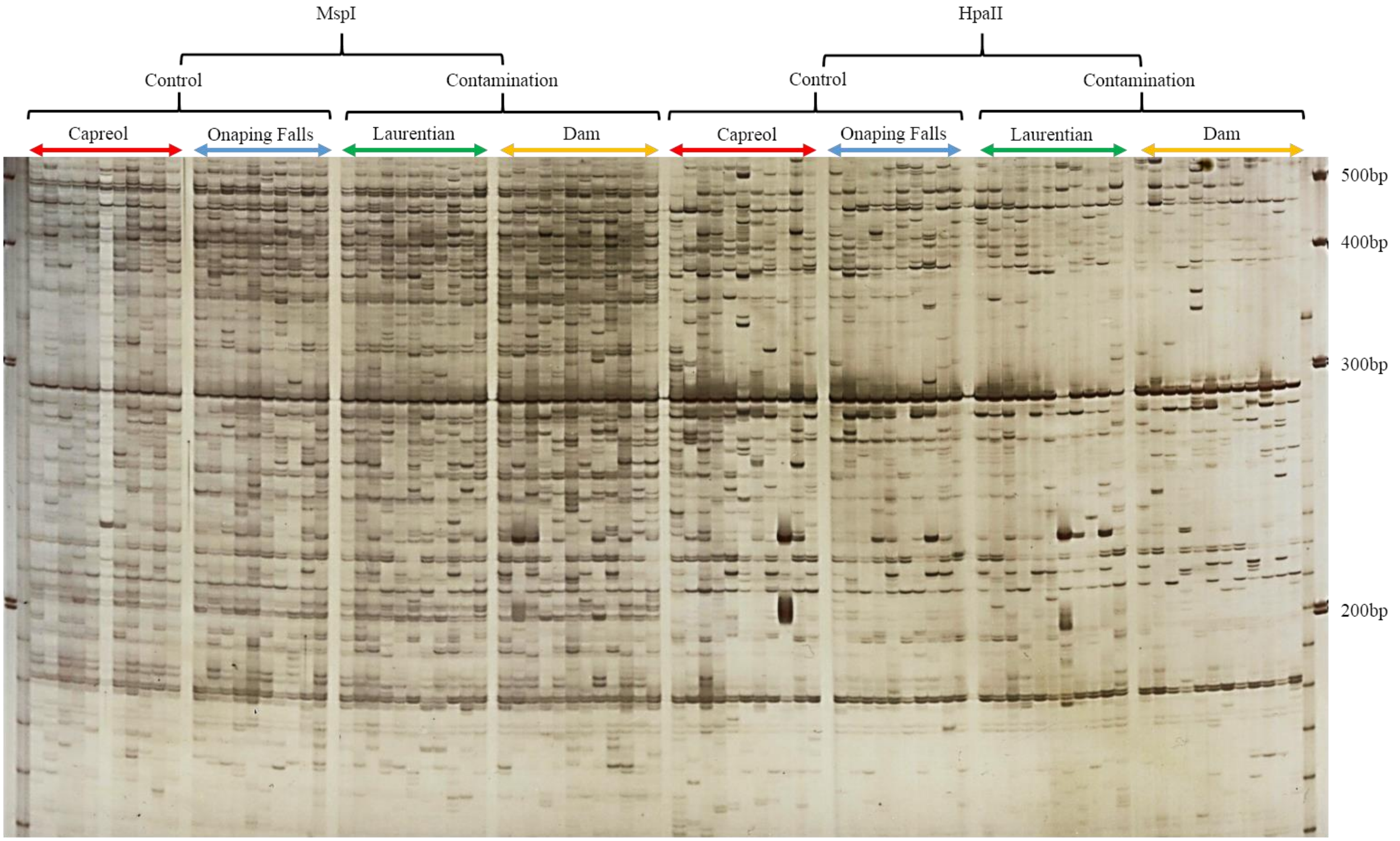

Supplementary Figure 2b

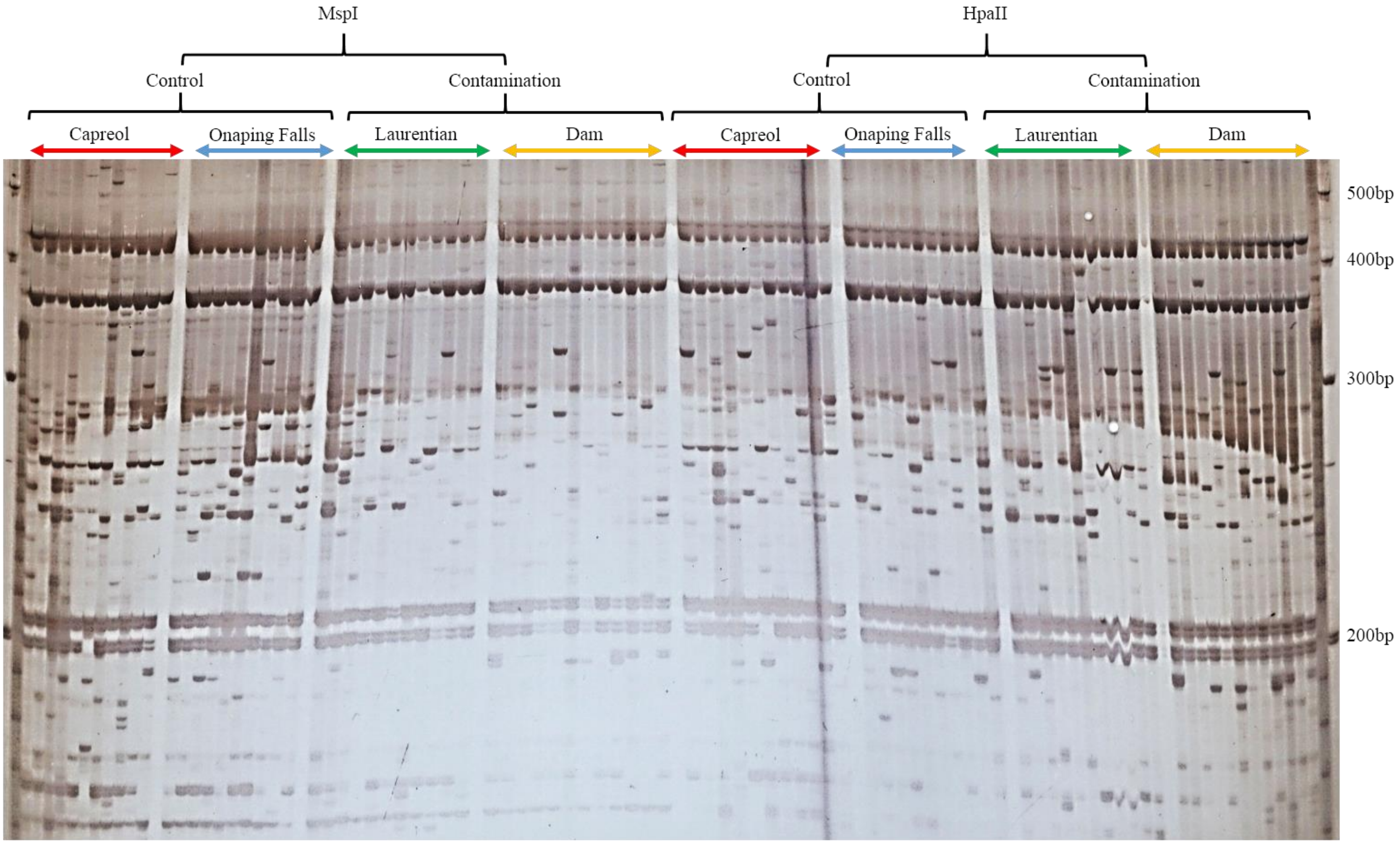

Supplementary Figure 2c

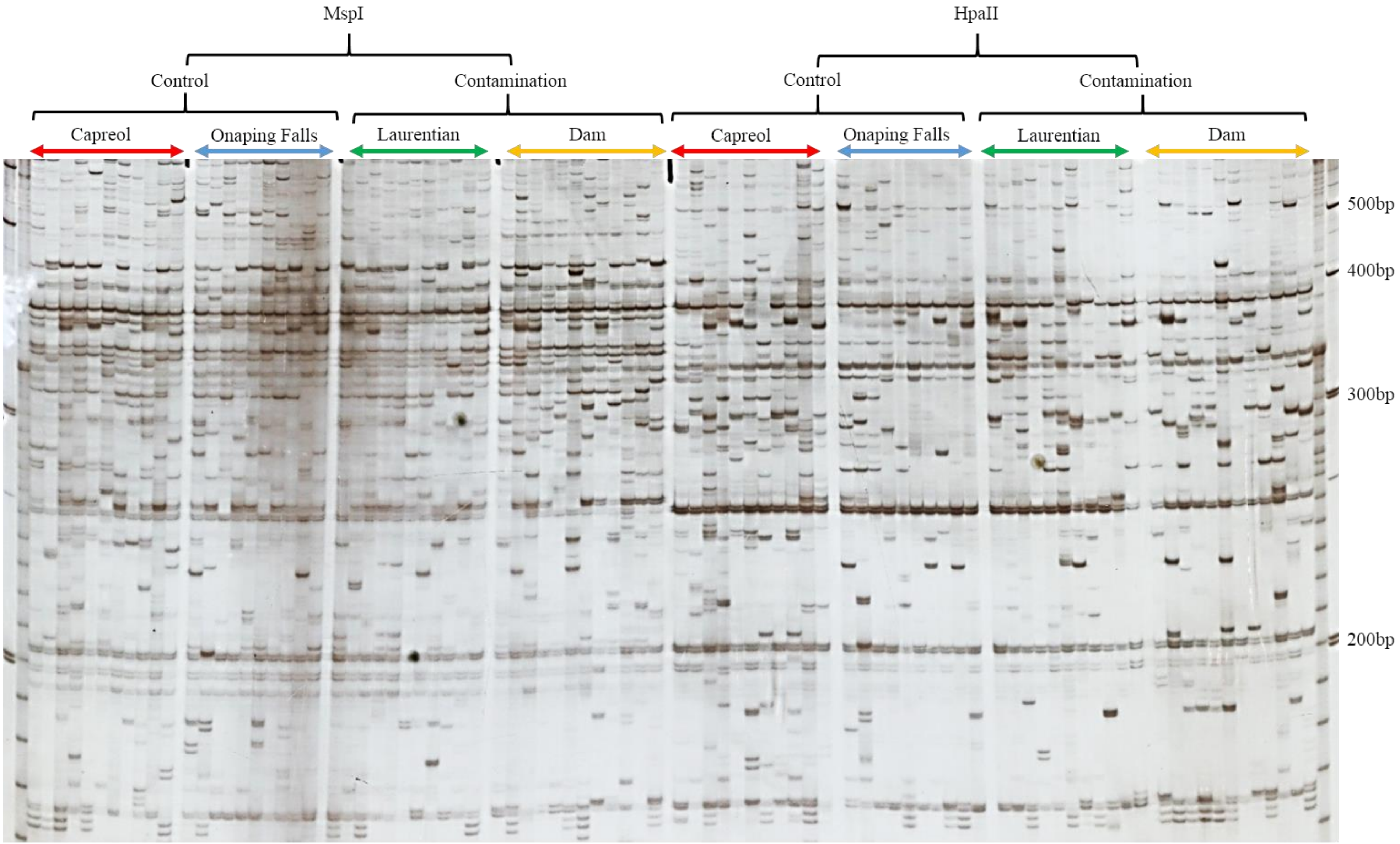

Supplementary Figure 2d

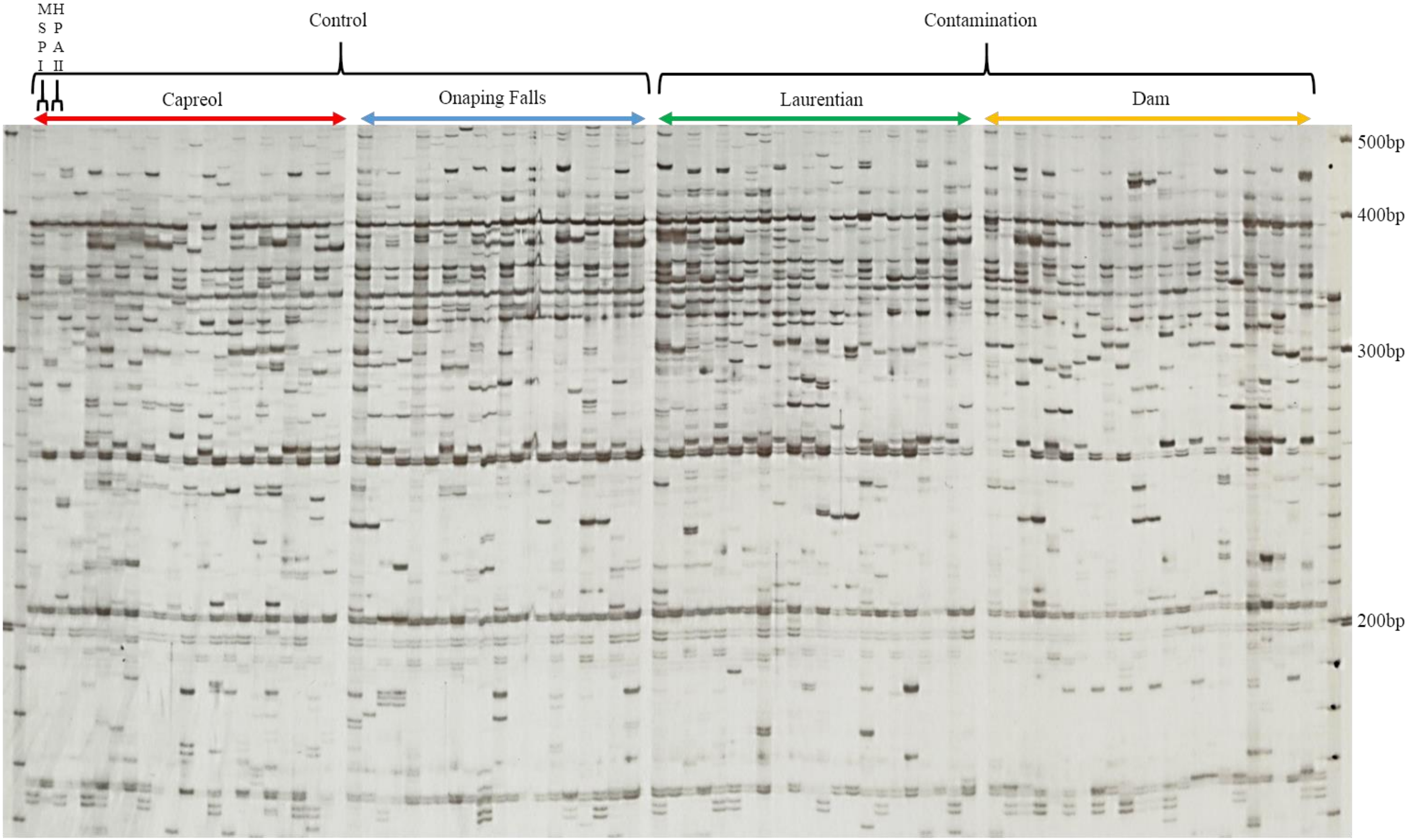

Supplementary Figure 2e

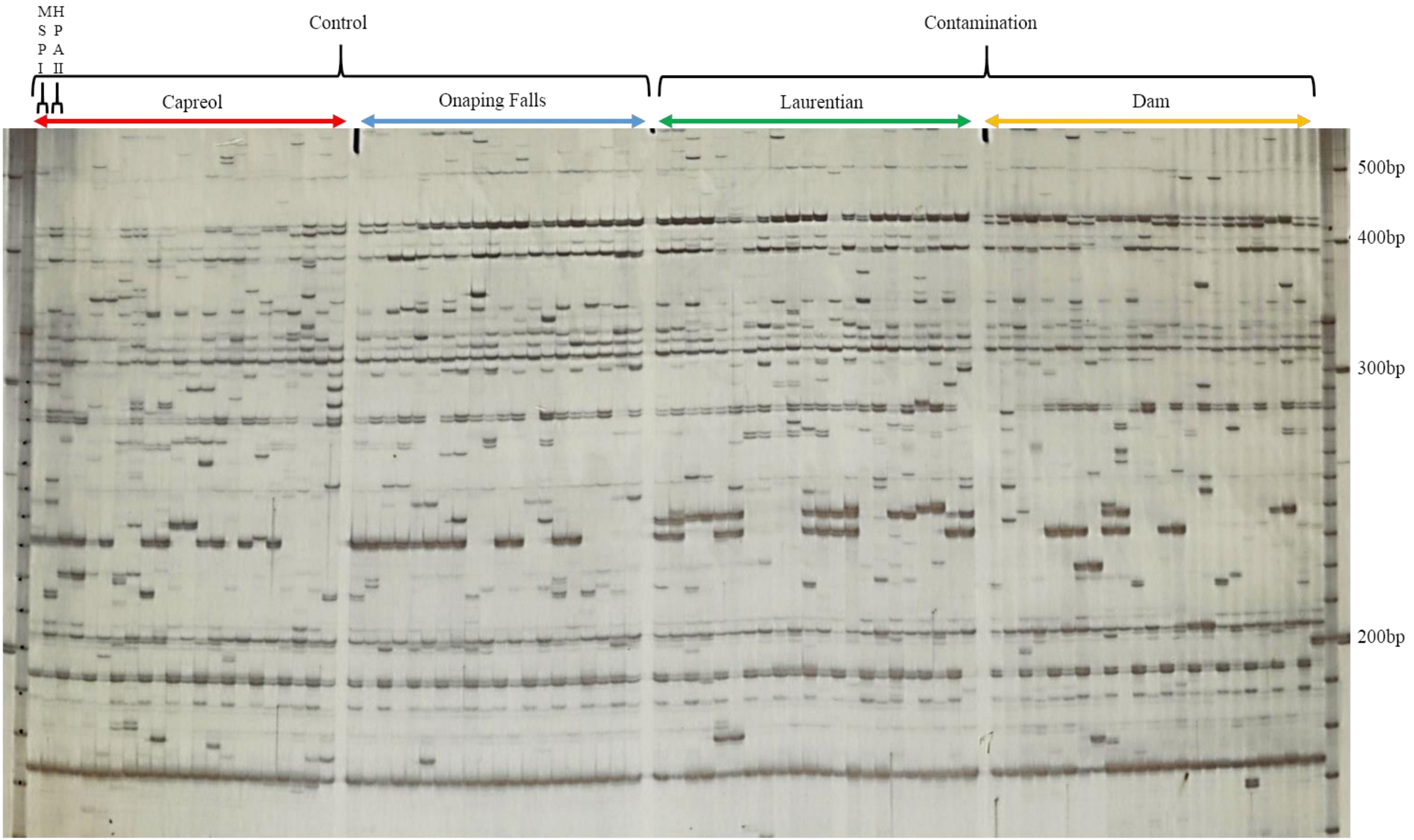

Supplementary Figure 2f

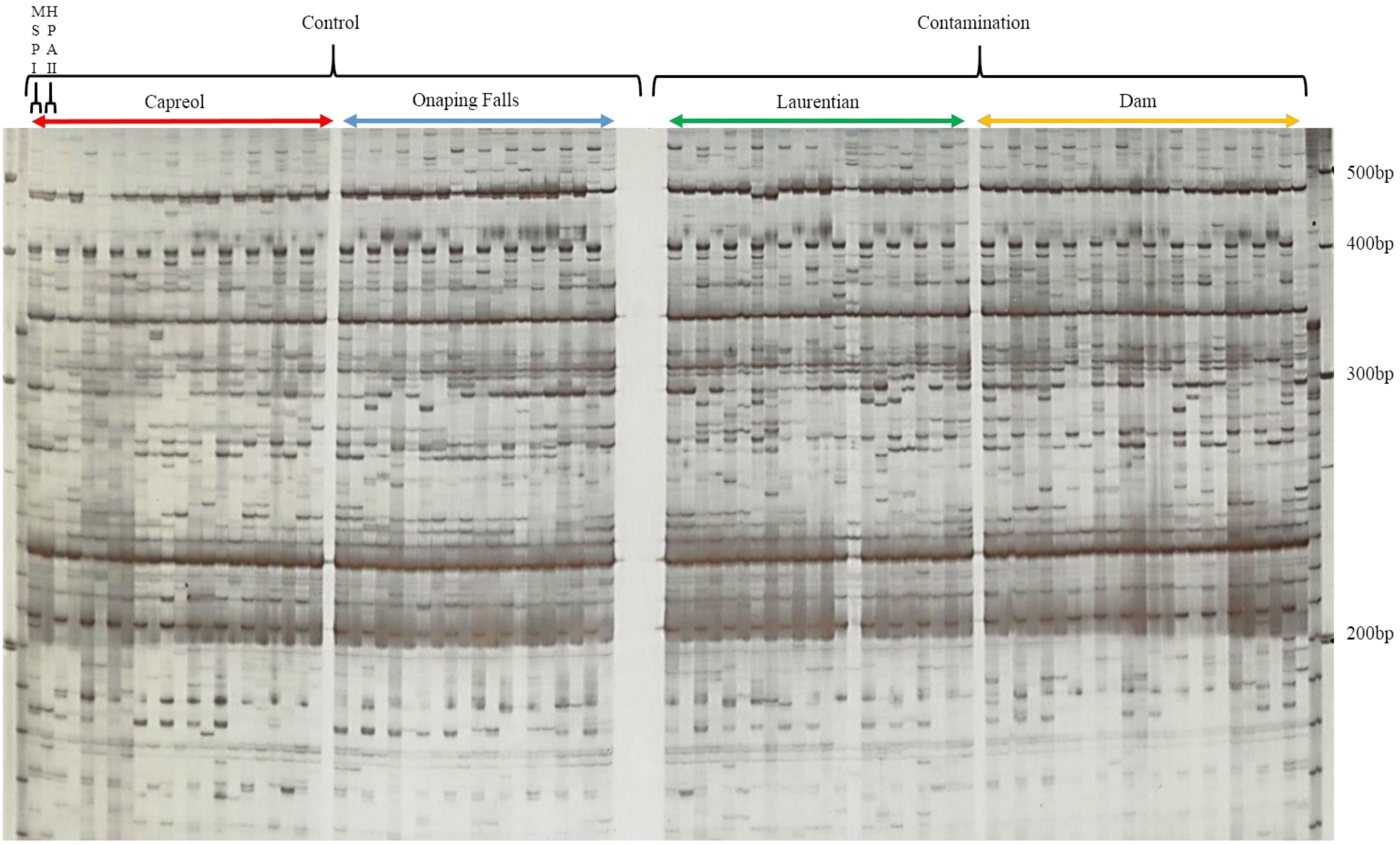

Supplementary Figure 2g

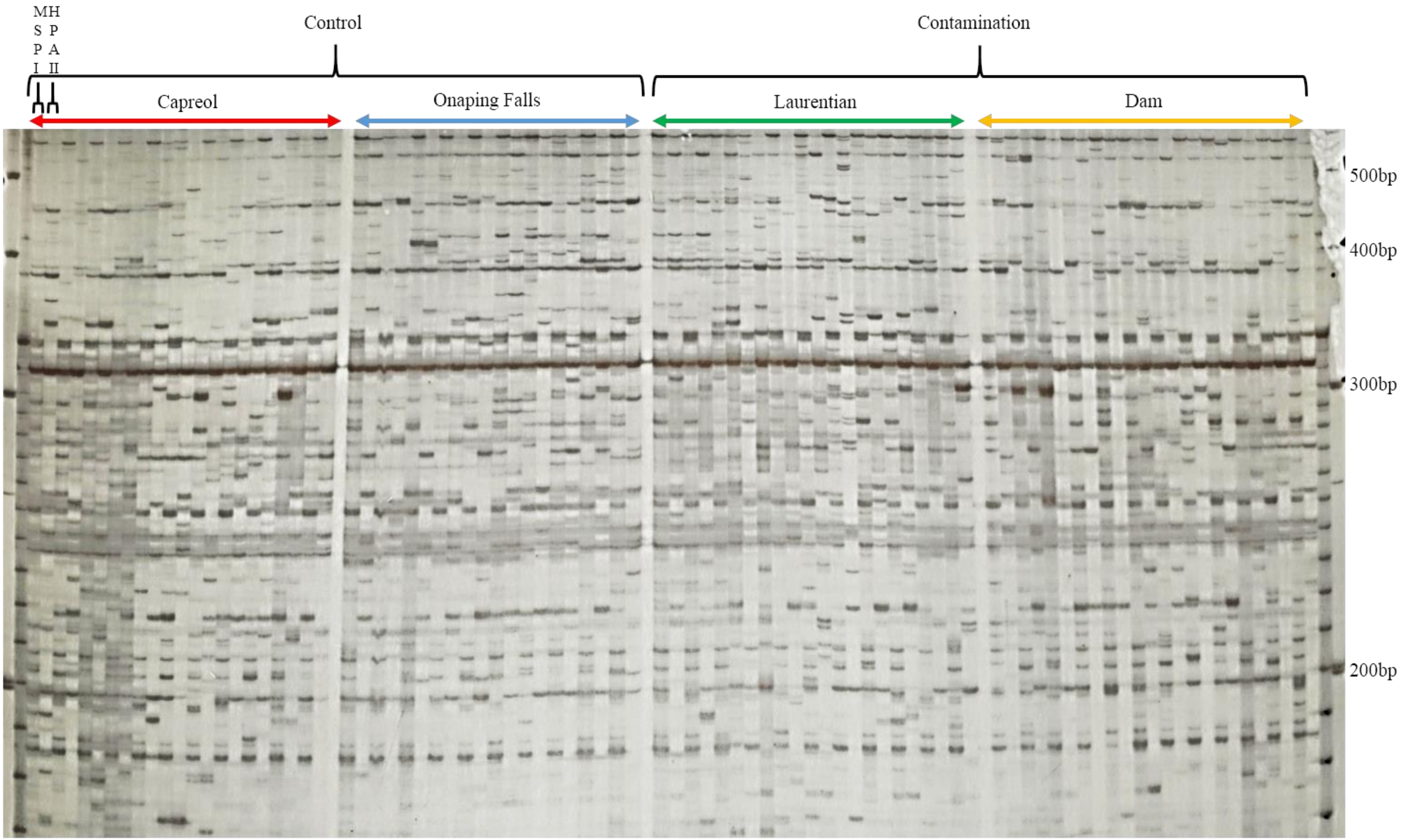

Supplementary Figure 2h
